# Modelling the current fractional cover of an invasive alien plant and drivers of its invasion in a dryland ecosystem

**DOI:** 10.1038/s41598-018-36587-7

**Published:** 2019-02-07

**Authors:** Hailu Shiferaw, Urs Schaffner, Woldeamlak Bewket, Tena Alamirew, Gete Zeleke, Demel Teketay, Sandra Eckert

**Affiliations:** 1 Water and Land Resource Centre, P.O. Box 3880, Addis Ababa, Ethiopia, and private P.O.Box, 7985 Addis Ababa, Ethiopia; 2 CABI, Rue des Grillons 1, CH-2800 Delémont, Switzerland; 3 Department of Geography & Environmental Studies, Addis Ababa University, P.O.Box, 1176 AAU, Ethiopia; 4 Botswana University of Agriculture and Natural Resources (BUAN), Department of Crop Science and Production, Private Bag, 0027 Gaborone, Botswana; 5 Centre for Development and Environment, University of Bern, Hallerstrasse 10, CH-3012 Bern, Switzerland

## Abstract

The development of spatially differentiated management strategies against invasive alien plant species requires a detailed understanding of their current distribution and of the level of invasion across the invaded range. The objectives of this study were to estimate the current fractional cover gradient of invasive trees of the genus *Prosopis* in the Afar Region, Ethiopia, and to identify drivers of its invasion. We used seventeen explanatory variables describing Landsat 8 image reflectance, topography, climate and landscape structures to model the current cover of *Prosopis* across the invaded range using the random forest (RF) algorithm. Validation of the RF algorithm confirmed high model performance with an accuracy of 92% and a Kappa-coefficient of 0.8. We found that, within 35 years after its introduction, *Prosopis* has invaded approximately 1.17 million ha at different cover levels in the Afar Region (12.3% of the surface). Normalized difference vegetation index (NDVI) and elevation showed the highest explanatory power among the 17 variables, in terms of both the invader’s overall distribution as well as areas with high cover. Villages and linear landscape structures (rivers and roads) were found to be more important drivers of future *Prosopis* invasion than environmental variables, such as climate and topography, suggesting that *Prosopis* is likely to continue spreading and increasing in abundance in the case study area if left uncontrolled. We discuss how information on the fractional cover and the drivers of invasion can help in developing spatially-explicit management recommendations against a target invasive plant species.

## Introduction

Invasive alien plant species (IAPS) are key drivers of anthropogenic global environmental change as they threaten native species, communities and ecosystems^1^, with significant consequences for the people living in the invaded range^2,3^. Yet, the management of widely established IAPS is often complex and challenging, as eradication is not feasible. Rather, management strategies may need to integrate multiple management options across the invaded range, considering the current geographic distribution and abundance of the target species, its environmental niche in the invaded region, the areas within the invaded range that are likely to experience high impacts, the distribution of high-value areas that should be kept free from invasion and the availability of funding for management^4–6^. Moreover, two classes of factors also affect management of IAPS: (i) intrinsic biological factors, such as the reproductive mode and the number of propagules produced^7^, and (ii) extrinsic environmental factors, such as dispersal vectors and invasion pathways^8^. Ignoring these factors may make the spatial management of IAPS challenging and complicated.

As management options may differ between areas with high IAPS cover and those areas where only isolated patches are found, accurate and detailed information is needed on the actual area invaded as well as on the local abundance or cover of IAPS across the invaded region. In recent years, an increasing number of papers have been published providing distribution maps of IAPS at different spatial scales and with different resolution^9^. One way to address this issue is to develop fractional cover maps of IAPS, but such mapping products of invasive species have rarely been developed^10^. Fractional vegetation cover assessments, mostly in semi-arid environments, were so far mainly a methodological research focus of the remote sensing research community. Machine learning algorithms, such as Random Forest (RF) in combination with satellite data, show the potential to model tree biomass successfully and accurately^11^ or to map a variety of vegetation covers^12^. Besides providing accurate predictions, they are able to model complex interactions among input variables, to identify most significant explanatory variables^13,14^, and to handle noise or outliers^15^. An approach with such advantages could possibly also be suitable for developing fractional cover maps as well as help identifying the relative importance of factors in explaining the current and predicting the future distribution and abundance of IAPS^13,14^, thereby providing important information for developing spatially explicit management strategies.

During the 19^th^ and 20^th^ century, various species and hybrids of the genus *Prosopis* that are native to South and Central America were planted in areas outside their native range (e.g. Australia, southern Asia, southern and eastern Africa) with the aim of providing firewood, charcoal, fodder and timber, to stabilize soil in degraded ecosystems, and prevent desertification^16^. The first introductions in Ethiopia were made during the 1970s and 1980s^17,18^, but problems arose soon thereafter, in the early 1990s, when the tree started invading croplands, grasslands, riverbanks and roadsides, thereby causing significant environmental impacts^19^, and becoming a source of conflict among pastoralist groups due to the resulting dwindling grazing land^20^. Despite its potential benefits, particularly regarding the provision of firewood and charcoal, Ethiopia has declared *Prosopis* a noxious weed and has recently published a national *Prosopis* management strategy^21^. In the case of *Prosopis*, various distribution maps have been developed with the goal to describe the spatial extent of *Prosopis* invasion^22,23^, its spread over time^23,24^, or model its potential invasion area^25^. However, all of these studies either focused on relatively small study areas or if they were extensive provided only coarse-resolution maps of either presence or absence of the species.

In this study, we set out to create a fractional cover map of *Prosopis* for the Afar Region, Ethiopia, one of the areas in Eastern Africa most heavily invaded by *Prosopis*. Most introductions in Ethiopia were made with *Prosopis juliflora* (Sw.) DC^20^, but as the taxonomic status of the invasive trees across study region has yet to be confirmed, we refer to the target species hereafter as *Prosopis*. The specific objectives of the study were (i) to assess the current distribution and cover of *Prosopis* invasion in the Afar Region by applying a robust machine learning algorithm (the random forest modelling) based on a large number of field data on local cover, and (ii) to identify factors that explain the current distribution of *Prosopis* along a cover gradient and drivers of future *Prosopis* invasion in the Afar Region. Specifically, we aimed to assess whether a) combining image spectral reflectance and environmental variables results in a better performance of the *Prosopis* distribution model than either group of variables alone, b) the overall distribution of *Prosopis* and the distribution of highly invaded areas is explained by the same or different variables, and whether c) landscape structures such as watercourses or road networks and environmental variables are suitable as predictors of future *Prosopis* invasion. Based on our findings, we outline approaches for using fractional cover maps and what they reveal about an invasion process to design and prioritize *Prosopis* management in this dryland ecosystem.

## Materials and Methods

### Study area

**Figure 1:**
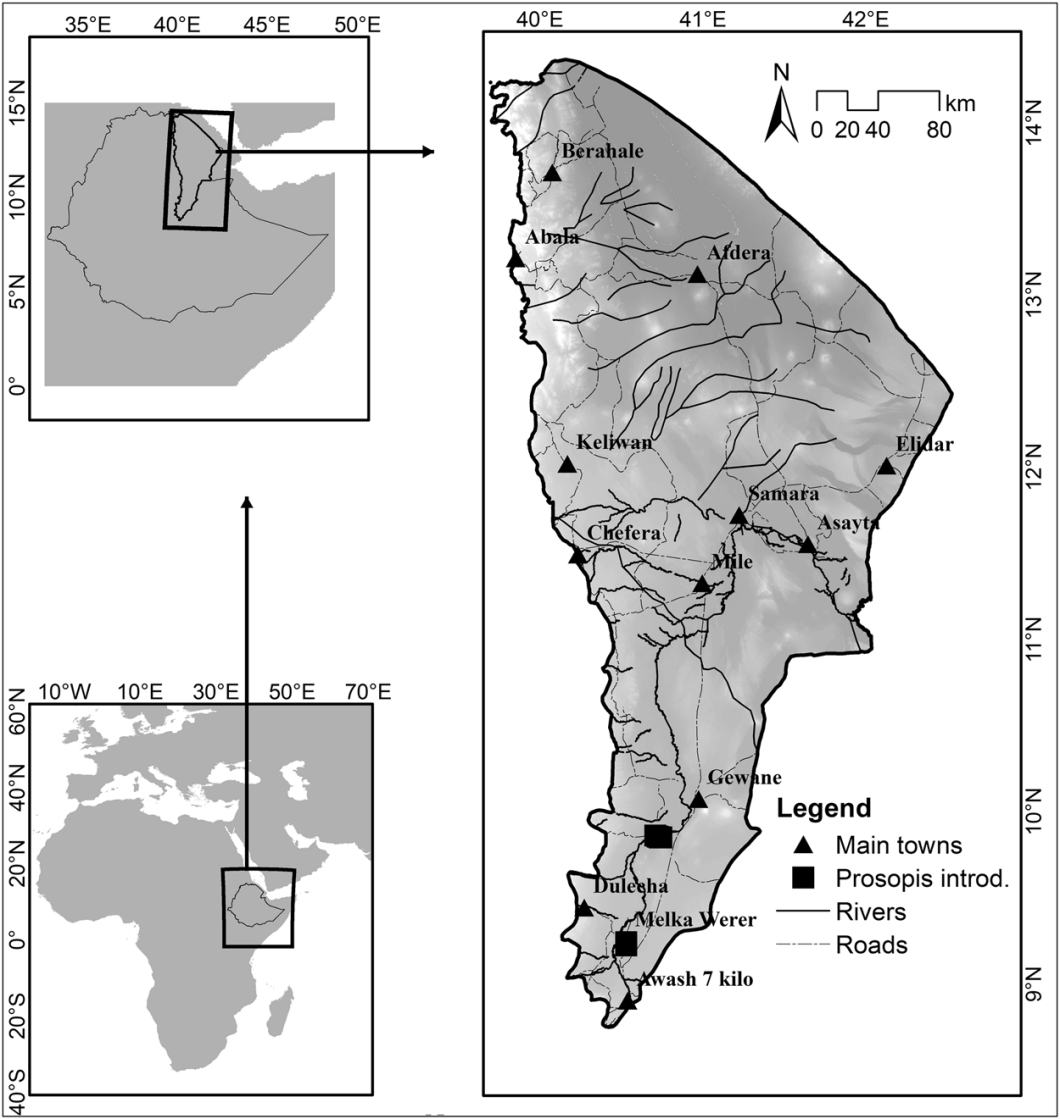
Location of the study area.

The study was carried out in the Afar Region, Ethiopia, which is part of the Great Rift Valley of East Africa. The study area extends from 8.8°N to 14.5°N and 39.7°E to 42.4°E, covers an area of 9.51 million ha and includes five zones, 30 districts and 376 kebeles (the lowest administrative units in Ethiopia; Fig. 1). The Afar Region has a mean annual rainfall of about 560 mm while the estimated mean evapotranspiration is 2,200 mm per year^26^. The region is the hottest part of Ethiopia, and the mean annual temperature is 31 °C. The mean maximum temperature reaches 41 °C in June and July, and the mean minimum temperature ranges from 21 to 22 °C between November to January. The mean monthly minimum and maximum rainfall recorded for the period 1965–1999 is 3 mm (June) and 121 mm (August), respectively, and the mean annual relative humidity ranges from 38 to 58 percent^26^. The major watershed in the Region is the Awash River Basin. Most parts of Afar Region are characterized by patches of scattered dry shrubs, acacia woodland, bushland, grassland and wooded grassland^27^.

The Afar people depend on the floodplains of Awash River for grazing during the drought periods and for small-scale agriculture^28^. Pastoralism is still the dominant source of livelihood for the Afar people, but agro-pastoralism is promoted by the government. In addition, governmental and private investments have led to large-scale agricultural production in the Middle Awash Valley, particularly in cotton and sugar cane production. Also, parts of the arable land have been abandoned as a result of soil salinity, which resulted from both inappropriate irrigation practices and high evapotranspiration of *Prosopis* invasion^28^.

### Study species

The genus *Prosopis* belongs to the family Fabaceae, subfamily Mimosoideae. It includes about 45 species, mainly found in the arid and semi-arid regions of the world^29^. *Prosopis* trees are able to fix nitrogen and have deep root systems, rendering them resistant to droughts. Several different species had been introduced into Eastern Africa and hybridization among parental species has been documented^16^.

According to Kebede and Coppock^20^, *Prosopis* was first introduced and planted in Ethiopia in the late 1970s at a nursery in West Harerghe and subsequently transplanted into the Afdem and Afar areas (Amibera and Gewane districts) in the early 1980s. Additional plantations were made between the 1980s and 1990s^20^ as shade trees in villages, around crop fields or to reduce erosion in degraded areas^23^. The locations of the original plantations reported by local key informants are compiled and shown in Figure 1.

### Sampling design and datasets

Georeferenced field data were collected throughout the entire study area using a stratified random sampling approach. Presence and absence plots were purposively selected from invaded and uninvaded areas, respectively. Invaded areas were additionally stratified into areas likely to be highly invaded (around villages, along roads, on riverbanks) and areas likely to be less invaded (outside villages, away from roads and riverbanks). The sampling plots were randomly selected within the different strata and substrata. In order to reduce spatial autocorrelation, sampling plots were selected that had a minimum separation distance of 500 m^30^. A total of 2,722 field sample plots of 20 m by 20 m (presence and absence plots) were collected between September 2016 and March 2017. A plot was considered a presence plot if it contained at least one *Prosopis* plant; otherwise it was considered an absence plot. About 70% (n = 1922) of the samples were absence plots and about 30% (n = 800) were presence plots. These proportions approximately correspond to the proportions of uninvaded and invaded land in the study area to avoid any bias of results towards either absence or presence of *Prosopis*^31,32^. Each geo-referenced sampling plot was recorded together with its level of *Prosopis* coverage, which was estimated visually to the nearest 10%. About 80% of all sampling plots were randomly selected to be used for model calibration and the remaining 20% were used for validation^33^.

The spatial datasets (17 independent variables) that we used to explain *Prosopis* distribution were gathered from various sources (Table 1). They differed in terms of spatial resolution^34^, projection, and time of acquisition; nearest neighbor spatial resampling and reprojection was applied to obtain a common spatial pixel resolution of 15 m. The field plots and the ancillary datasets (explanatory variables) had relatively equivalent pixel size^35^. Spatial resampling was done to match the dataset with the highest spatial resolution, the panchromatic band of the Landsat 8 Operational Land Imager (OLI) data was used in this study. In total, nine scenes were needed to cover the study area. They were acquired by the Landsat 8 (OLI) on 26 and 28 January as well as 11 and 20 February 2017 (paths: 167 and 168; rows: 50 to 54). These acquisition dates match the period of field data collection and fall into the study area’s dry season, when herbs and grasses are dry and most trees and bushes except *Prosopis* have shed their leaves. The remotely sensed datasets were visually checked for geometric correspondence to all other datasets. Further, these datasets were atmospherically corrected using the Landsat Ecosystem Disturbance Adaptive Processing System (LEDAPS) algorithm^36^. We selected the red, the near-infrared and the first shortwave-infrared bands as explanatory variables among image reflectance^37^. Furthermore, we calculated the normalized difference vegetation index (NDVI), which is a ratio of the difference between the near-infrared and the red band to the sum of the near-infrared and the red band of a specific sensor. All selected bands as well as the NDVI have proven suitable for capturing photosynthetic active vegetation, and soil and vegetation moisture content^37^. Additionally, we included two products of the MODIS sensor in our analysis, daytime (LSTd) and nighttime (LSTn) land surface temperatures. In order to have longer term LSTd and LSTn average data we calculated 5-year averages from the monthly LSTd and LSTn products that were generated between 2012 and 2017. The spatial resolution of these data is 1 km. Although this seems rather low compared to the other datasets, these day- and night-time temperature datasets have been shown to be useful in species distribution modelling^38^. We furthermore sought to understand if day- and night-time temperatures are important explanatory or drivers of *Prosopis* distribution. Additionally, we used variables representing topography, infrastructure, and watercourses. These variables have been previously shown to influence distribution of invasive species^39,40^.

**Table 1:** Variables used to explain *Prosopis* distribution and abundance in the Afar Region.

Variable abbreviations	Description	Source
Rain	Mean annual rainfall	Ethiopian National Meteorol. Agency
Temp	Mean monthly temperature	Ethiopian National Meteorol. Agency
LSTd	Monthly land surface temperature during daytime and nighttime; for the modelling 5-year averages were calculated	MODIS, NASA
LSTn	Monthly land surface temperature during nighttime; for the modelling 5-year averages were calculated	MODIS, NASA
PAN	Panchromatic reflectance	Landsat 8 OLI, USGS
Red	Red reflectance	Landsat 8 OLI, USGS
NIR	Near-infrared reflectance	Landsat 8 OLI, USGS
SWIR1	Shortwave infrared band 6 reflectance	Landsat 8 OLI, USGS
NDVI	Normalized difference vegetation index	Calculated from Landsat 8 OLI, USGS
Elevation	Shuttle Radar Topography Mission digital elevation model (30 m spatial resolution)	USGS
Slope	Derived from Elevation	Calculated from Elevation
Relief	Derived from Elevation	Calculated from Elevation
Landform	Derived from Elevation	Topographic position index derived from elevation, aspect and slope
Rugged	Derived from Elevation	Ruggeddeness index calculated from Elevation
DistRoad	Distances derived from road network data	Ethiopian Road Authority
DistVillage	Distances derived from settlement data	Calculated from EthioGIS and Central Statistical Agency
DistRiver	Distances derived from data on watercourses	Calculated from EthioGIS

MODIS: Moderate Resolution Imaging Spectroradiometer; NASA: National Aeronautics and Space Administration; USGS: United States Geological Survey.

### Random forest modeling

To regress and assess fractional cover and distribution of *Prosopis* in the Afar Region, we used Random Forest (RF), a nonparametric technique based on classification and regression trees (CART)^13^. RF consists of an arbitrary number of trees, where each tree is generated by bootstrap samples. About one third of the overall sample is left for validation (the out-of-bag predictions – OOB). Each split of the tree is determined using a randomized subset of the predictors at each node. The final outcome is the average of the results of all the trees^13,14^. The number of decision trees was found to be optimal if set to 5,000 trees. The number of features was set to the square root of the number of input variables, as has been done in another study^41^. We carefully checked that the out-of-bag error was stable with the chosen settings before applying the model. We assessed the model performance by calculating its accuracy, Kappa coefficient, correlation, and the Receiver Operating Characteristics (ROC) of area under the curve (AUC). It is a prevalence- and threshold independent measure of model performance suitable for evaluating the performance of ordinal score models^42^. Moreover, we calculated sensitivity (true positive rate - TPR) and specificity (true negative rate - TNR) of the model^43^.

We applied a 10-fold validation approach, i.e. repeating the validation in ten different calibration batches and comparing the outcome with that of the training data for each of these batches^44^. We then calculated the threshold value for presence and absence of *Prosopis*^31,39^. This approach identified the minimum area where *Prosopis* trees occur whilst ensuring that no localities at which the species has been observed are omitted, i.e. omission rate of the minimum^39^ with a certain precision obtained from the model.

### Factors explaining the current distribution of *Prosopis*

To achieve greater stability of the models, we first assessed the importance of variables in each model by using the method described by Natekin and Knoll^45^. We then removed variables from the final models if they contributed less than 5% to the model (Appendix 1). To assess the importance of the various sets of explanatory variables in explaining the overall distribution of *Prosopis* and the distribution of areas with increasing *Prosopis* cover (i.e. areas with potentially increasing environmental impacts), we set two types of iterations. Iteration (i) used three scenarios with different sets of explanatory variables: scenario 1 included all image spectral reflectance and environmental variables (Appendix 1); scenario 2 included spectral reflectance variables only, and scenario 3 included environmental variables only. Iteration (ii) used scenario 1 but with four different threshold levels of presence input cover estimations, i.e. at ≥5%, ≥20%, ≥40%, and ≥60%.

We used a weighted mean fitted analyses approach^46^ to explore the main environmental correlates of the current *Prosopis* distribution and cover^40,46–48^. This approach provides more consistent information on the shape of the fitted surface than what can be obtained from individual functions with respect to the species distributions^49^.

### Drivers of *Prosopis* invasion

To identify the main drivers of the invasion of *Prosopis*, we determined the relative importance of input variables. As the use of multivariate regression requires standardization (centering and scaling) to identify each variable’s influence on species distribution^50,51^, we standardized the variables by subtracting means from each corresponding value of the driver, and dividing the center value by the standard deviation^51^. In the interpretation of potential drivers of *Prosopis* invasion, we excluded the spectral variables NDVI, NIR and SWIR that all respond to vegetation photosynthetic activity, since this spectral response can be considered both as cause and effect of *Prosopis* presence.

### Statistical analyses

All model calculations and validations were implemented in the open source R software version 3.3.3, while mapping of outputs were carried out using ESRI ArcGIS. The RF modelling was implemented in R using the randomForest^52^ and boosted regression tree (brt) packages^53^. The relative importance of the explanatory variables was estimated using the Friedman formulae^54,55^ which is contained the gbm package^47^. Moreover, the contribution and direction of importance of each variable were evaluated by analyzing their marginal effect curves and fitting polynomial smoothing^47,48^. Finally, the fractional cover gradient of the RF model output was matched with the ground invasion level by correlating the threshold levels of presences and absence.

## Results

### Extent of invasion for the current distribution of *Prosopis*

**Figure 2:**
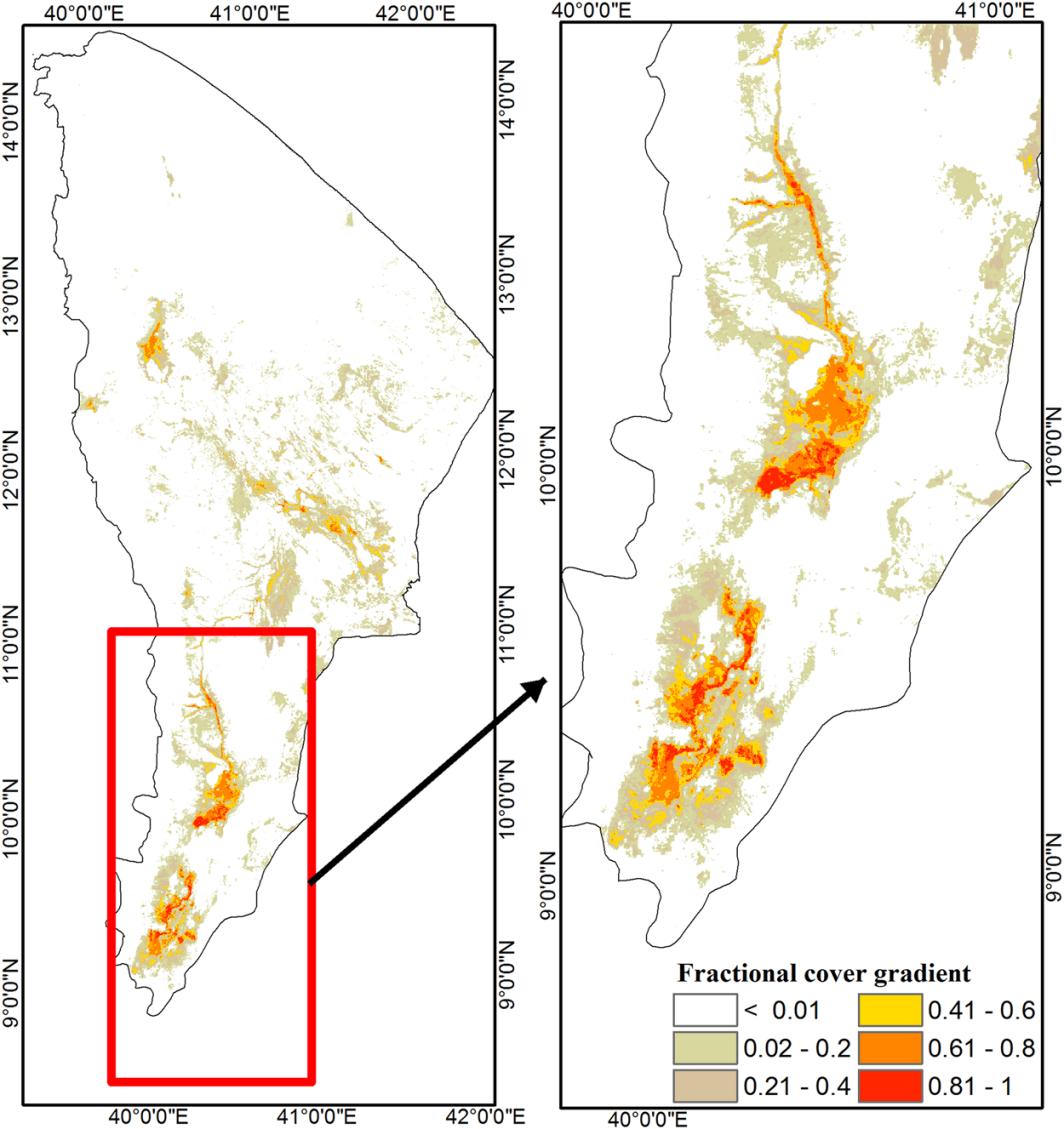
Fractional cover gradient map for *Prosopis* distribution and abundance in the Afar Region of Ethiopia. (**A**) Regional level fractional cover gradient (0–100%), and (**B**) fractional cover zoomed into *Gabi Rasu* Zone.

The area invaded by *Prosopis* in the Afar Region is estimated to be about 1,173,000 ha (12.3% of the total regional area; Fig. 2, Appendix 2). Validation of the model performance revealed an accuracy of 92% and a Kappa coefficient of 0.8 at P < 0.001 (Table 1). In addition, sensitivity and specificity evaluations were 0.894 and 0.926, respectively, and AUC, correlation and threshold measures were also high (Appendix 1). The model output threshold was identified as 0.326, which corresponds to the minimum starting cover level of ~0.004% of *Prosopis* presence map at 15 m pixel size.

By 2017, four of the five zones and 21 of the 30 districts in the Afar Region were invaded by *Prosopis* (Fig. 2A; Appendix 2). *Prosopis* distribution was the highest in Zone 3 (Gabi Rasu), with 193,920 ha in Gewane district (22% invaded) to 74,972 ha in Bure Mudaytu (68% of area invaded; Fig. 2B, Appendix 2). Districts with high mean cover were identified from different zones: in Zone 3 Amibara District (32%), in Zone 5 Artuma District (24%), in Zone 4 Teru District (23%) and in Zone 1 Aysaita District (23%). Only 9 districts were found to be almost free of *Prosopis*, i.e. Berahle, Erebti, Megale, Afdera, Dalul and Abala districts in Zone 2, Argoba Special and Koneba districts in Zone 3, and Gulina District in Zone 4. By 2017, all of these districts had less than 1.3% of their area invaded by *Prosopis* (Appendix 2).

### Explanatory variables for the current distribution of *Prosopis*

**Figure 3:**
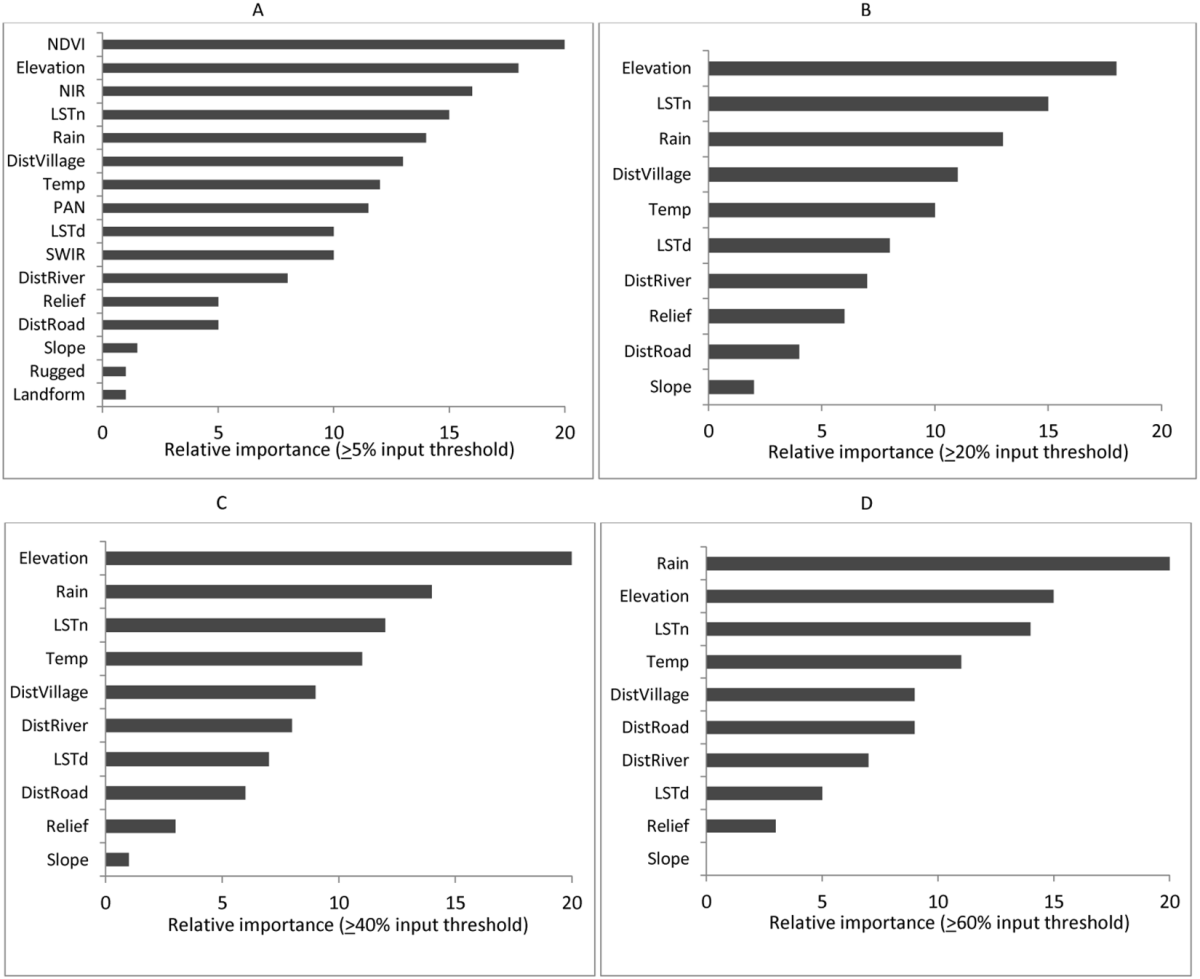
Relative importance (%) of explanatory variables at different tested scenarios of input cover threshold levels: (**A**) ≥5%, (**B**) ≥20%, (**C**) ≥40%, and (**D**) ≥60%.

Both the selection of explanatory variables and input threshold levels of visual estimated cover affected model performances. Among the three tested scenarios, scenario 1 generated the highest performance relative to scenarios 2 (using image reflectance variables only) and 3 (using environmental variables only; Fig. 3). NDVI, Elevation and NIR were the three most important variables for scenario 1 (Fig. 3A). In scenario 2, SWIR, NIR, NDVI were ranked highest, and in scenario 3 Elevation, LSTn, and Rainfall (Appendix 1).

Shifting the input threshold levels for *Prosopis* cover in scenario 1 from ≥5% to ≥60% had only moderate effects on the ranking of the explanatory variables. NDVI revealed the highest relative importance independent of the input threshold, followed by Elevation, DistVillage and DistRiver. The only consistent change with increasing input threshold for *Prosopis* cover was an increasing relative importance of DistRoad (Fig. 3).

**Figure 4:**
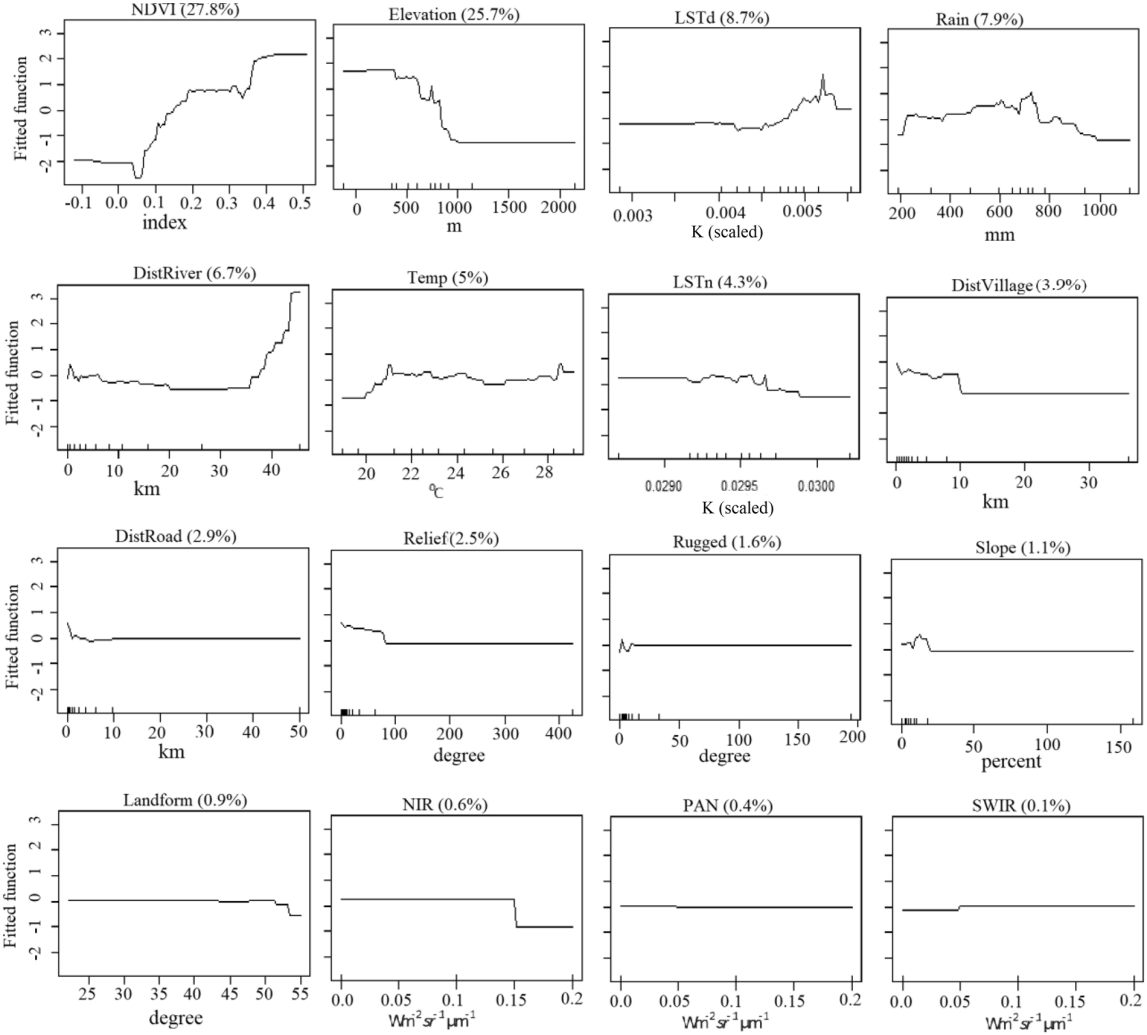
Partial dependency or marginal effect curve indicating overall importance of 16 variables for explaining *Prosopis* (the number in brackets next to each variable name is the proportion of *Prosopis* fractional cover explained by that variable in final model) (y-axis for all plots are fitted values, but x-axis are different units).

Individual explanatory variables differed in terms of the spatial range of interaction with the dependent variable, *Prosopis* presence. DistVillage affected *Prosopis* presence up to a distance of 10 km, while DistRiver and DistRoad mainly affected *Prosopis* presence over a distance of a few hundred to thousand meters (Fig. 4). Environmental correlates of *Prosopis* distribution and cover are indicated by the magnitude and direction of weighted mean fitted values of each explanatory variable in relation to the response variable (fractional cover). Important environmental correlates of the current *Prosopis* distribution and cover were DistRivers, DistRoads and DistVillage, with average values of distances up to 5913 m, 2679 m and 1937 m, respectively (Appendix 3). *Prosopis* is currently preferentially found at an altitude of below 1000 m (average 585 m asl) and an mean annual temperature of 20–29 °C (average 24.4 °C). Annual rainfall of 200–1000 mm (average 608 mm) was most conducive for the current distribution and cover of *Prosopis*, but some invaded areas experience less than 200 mm rainfall (Fig. 4).

### Drivers of *Prosopis* invasion

**Figure 5:**
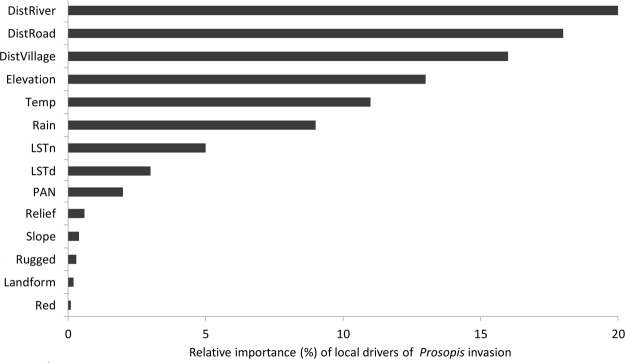
Standardized relative importance of predictors (drivers) of *Prosopis* invasion in the Afar Region.

The three landscape variables DistRiver, DistRoad, and DistVillage were the most important drivers of local *Prosopis* invasion (Fig. 5). In contrast to their role in explaining the current distribution of *Prosopis*, climatic variables appear to be only moderately important drivers and image reflectance variables are to be the least important drivers of invasion.

## Discussions

### Extent of invasion for the current distribution of *Prosopis*

Within approximately 35 years, *Prosopis* has invaded about 12.3% of the land in the Afar Region of Ethiopia. It is most widespread in the southern parts of the Afar Region, especially in Zone 3. In the areas where the original plantations had been made, *Prosopis* has already invaded an average of up to 31% of the district’s area in Amibara (with 32% cover abundance) and about 68% of the land was invaded in Bure Mudaytu District (with 30% cover abundance). The southern part of the Afar Region is not only the point of introduction of *Prosopis* but it has also experienced many economic activities over the last four to five decades^34^ that could contribute to spread of the tree. For instance, large government-owned investments, private farms, and populations of pastoralists, and agro-pastoralists are found in the southern part of the Region^23^. Hence, habitat disturbance due to land-use changes^23^ may further explain why *Prosopis* invasion is more pronounced in the southern than the northern parts of the Afar Region.

In contrast to previous studies^25^, our fractional cover map indicates large areas in the Afar Region with low *Prosopis* densities, and some of these areas are relatively far away from the original plantations. This suggests that *Prosopis* invasion in the Afar Region is likely to continue increasing over the next decades if no measures to manage this invasive species are implemented.

### Explanatory variables for the current distribution of *Prosopis*

By combining Landsat 8 image reflectance variables with variables describing the topography, climate and landscape structure of the Afar Region (scenario 1), we achieved approximately 92% accuracy for modelling the fractional cover of *Prosopis* across the invaded range. Modelling the cover of *Prosopis* using either image reflectance variables only or environmental variables only resulted in a lower accuracy, suggesting that the integration of image reflectance and environmental variables was responsible for the highest accuracy of the model used for mapping the fractional cover of *Prosopis* in Afar Region. Other studies developing invasive plant species distribution models also found that adding generic variables such as climate, land use and disturbance increased the accuracy of the models^1,30^.

Our results suggest that *Prosopis* started to spread from multiple focal points (original plantation sites, villages, etc.) and subsequently invaded the surroundings along linear corridors (river courses, travel routes) that act as dispersal pathways. Linear landscape characteristics have been repeatedly shown to explain IAPS distributions^56,57^. Bartuszevige^57^ found that towns as focal points were also of primary importance in explaining the distribution of the invasive shrub *Lonicera maackii* in the eastern U.S.A., and secondary invasion of *L*. *mackii* to suitable habitats was mediated through birds. Similarly, invasion of *Prosopis* into areas away from DistVillages, DistRivers or DistRoads appears to occur at a lower cover, but the role of additional factors affecting the current distribution or cover of *Prosopis*, such as migration corridors of livestock and wildlife, warrant further investigations as they could be used as ‘jump dispersal’ agents^58^.

In an attempt to explore the relative importance of factors of invasions of three woody IAPS in South Africa, Rouget and Richardson^59^ found that geology, climate, land use, and topography adequately explained distribution of these species, while cover was better explained by propagule pressure, measured as distance from putative invasion foci. Considering that variables such as DistVillage, DistRiver and DistRoad can also be interpreted as indicators of dispersal pathways, the highest ranking of DistRiver and DistVillage in the models with and without reflectance data suggest that propagule pressure plays an important role in explaining both the current distribution and cover of *Prosopis* in the Afar Region.

The importance of DistRoad and DistRiver on *Prosopis* invasion extended up to 10 km at both sides in a non-linear relationship. However, in most parts of the study area, the highest invasion of *Prosopis* occurred within a strip of ten to a few hundred m along roadsides and riverbanks. Similarly, Flory and Clay^60^ showed that densities of three invasive species had higher germination rates than natives in USA, declined with increasing distance to the nearest road, and the percent cover of exotic species was significantly greater at 10 and 100 m from roads than at 1000 m from roads^61^.

Partial dependency or marginal effect curve indicated that NDVI had the highest contribution to explain the current distribution of *Prosopis* in the arid ecosystems (Fig. 4) because it is an effect of invasion^46^. Moreover, weighted fitted mean values of independent variables (Appendix 3) elucidate the importance of interactions between predictors and identify the optimal habitats of species^49^. It should be noted, however, that areas with high *Prosopis* cover are usually areas that were colonized first (Shiferaw *et al*., unpubl.), suggesting that large areas currently with low cover will increase in cover within the next decades.

### Drivers of *Prosopis* invasion

Decision tree-based regressions such as RF have been recently proposed for predicting invasion processes of IAPS^8,62,63^. For example, Menuz and Kettenring^8^ found that climate variables were most important for predicting invasive plant species distributions across the large study area and factors related to nutrients, land cover, and disturbance had only moderate importance. Starting with a climate model and then nesting models with additional landscape, land use and structural predictors, Vicente *et al*.^64^ explored a range of different drivers of plant invasions in Portugal. They found that climate was the most important driver, while other landscape variables contributed where climate was favourable.

Our study suggests that, among the variables included in the analysis, river courses, road networks and village vicinities are the most important drivers of *Prosopis* invasion in the Afar Region (Fig. 5). River courses, particularly during flooding events, are known to serve as modes of transportation of *Prosopis* pods and seeds to downstream areas, a process termed ‘corridor dispersal’^58^. Besides transportation, seeds of *Prosopis* transported by river-courses also benefit from increased moisture that allows them to germinate and grow quickly. Similarly, ditches along road-networks maintain moisture longer than the surrounding ecosystems, thereby offering *Prosopis* ideal conditions for germination and early growth (Shiferaw, personal observation). In particular, our findings suggest that the invasion of the riparian habitats along the Awash River will continue downstream, thereby accelerating the loss of drought-season grazing areas and also aggravates associated conflicts among pastoralist communities^65^.

While invasion of *Prosopis* into areas away from villages, rivers or roads appears to occur at a lower rate, the role of additional factors driving invasion by *Prosopis*, such as migration corridors of livestock and wildlife, warrant further investigations. Pods of *Prosopis* have sugary flesh and contain several seeds which makes them important food sources of both wild and domestic animals during the long dry season^26^. Animals regurgitate, scratch or break hard seed coats, and scratched seeds germinate and grow faster than intact seeds, due to increased moisture imbibition and dung acting as manure^26^.

In contrast to Menuz and Kettenring^8^ and Vicente *et al*.^64^, our data suggest that precipitation and elevation, which are climatic variables describing the environmental niche of plant species^35^, are less significant drivers of future *Prosopis* invasion than are landscape structure variables, at the current stage of invasion. This supports our previous notion that a considerable part of environmentally suitable habitats in the Afar Region have not yet been fully colonized by *Prosopis* and that this plant invader is therefore likely to continue spreading and increasing in abundance if left uncontrolled.

### Fractional cover approach

Fractional cover mapping is a relatively recent approach to combine both information on the overall distribution and the variation in abundance or cover of an alien species in the invaded range. Fractional cover maps provide key information regarding the overall impact of IAPS, as they provide information on the overall invaded area and on local abundance^66^. As with many other invasive plant species, the impact of *Prosopis* on biodiversity and ecosystem processes has been shown to be abundance- or cover-dependent^67^.

Fractional cover maps have only rarely been used to assess the current distribution of IAPS and their abundance across the invaded range. Frazier and Wang^10^ assessed the spatial and local status of saltcedar (*Tamarix* spp.) invasion in parts of the invaded range in North America, and they suggested that saltcedar had originally invaded the region along river-courses but subsequently expanded away from the immediate riparian zones. They concluded that targeting isolated saltcedar stands from the riparian zone should be part of an integral saltcedar management strategy in the region.

Our approach to model the fractional cover of *Prosopis* in the Afar Region achieved an accuracy of 92% (with an AUC of 0.97), which is higher than accuracies achieved in most other species modelling approaches^25,68^. This is in agreement with previous studies, suggesting that RF is a suitable algorithm for species distribution modelling^8^ and fractional cover mapping^14^. Moreover, fractional cover mapping approach also allowed identification of isolated, small patches of *Prosopis* in the Afar Region, which are main targets for management (see below subheading). In this study, we converted the modelled probability maps into fractional *Prosopis* cover maps by setting the threshold of uninvaded areas to 0.326. We used the maximum intersection of sensitivity and specificity as threshold^31,39,48^.

We suggest that five points should be considered in order to achieve good results while applying RF regression: (1) sufficient and well-distributed field data samples should be collected in the study area; (2) the number of presence and absence field samples should be proportional to the overall proportions of sites in the study area where the species is present and absent, respectively; and (3) the field data values for the dependent variable should be within the range of the expected prediction values, (4) as shown by previous study, the values of explanatory variables used for training need to represent the entire range of values present in the study area^69^, and (5) variable reduction and fine-tuning of selected parameters can improve the model fitness and regression outputs.

### Fractional cover map and invasive species management

Understanding the extent of the current distribution and abundance of IAPS and predicting future invasion pathways are crucial for scientists and decision-makers who wish to quantify the regional impacts of IAPS and to design management strategies that help reducing the current cover levels and the further spread of IAPS^35,58^. As eradication is usually not possible for widespread IAPS, spatially explicit management strategies should be developed that assign different control objectives (prevention of spread to unoccupied areas, local eradication, containment and asset protection) to areas with different invasion level^6,70,71^. Recently, Ethiopia adopted a National Strategy on *Prosopis juliflora* management^21^. The strategy includes three main objectives: (i) prevent the expansion of *Prosopis* to uninvaded areas; (ii) reclaim and restore invaded areas after *Prosopis* clearance; and (iii) sustainably manage of *Prosopis* for productive use and increasing biodiversity through regulation and coordination of *Prosopis* management initiatives^21^.

Our *Prosopis* fractional cover map provides essential information for implementing an early detection and rapid response system, which is primarily targeted at areas with low *Prosopis* cover and at neighboring uninvaded areas. Prevention of permanent establishment of IAPS in uninvaded areas is a key component of most invasive species strategies^67^ as it is the most cost-effective means to prevent losses of ecosystem services due to ecosystem degradation^72,73^. Based on our findings, surveillance of uninvaded areas should particularly focus on buffer zones of approx. 10–100 m along roadsides and riverbanks, but may need to be extended to other linear structures such as livestock migration routes. If *Prosopis* is found within the buffer zones along linear structures, the area should be further surveyed up to one kilometer away from the linear structures (Fig. 4). Emphasis should also be put at targeting and managing isolated *Prosopis* patches since satellite populations of IAPS may play a key role in the future spread of IAPS^1,73,74^. The high resolution and accuracy rate of our *Prosopis* fractional cover map helped identifying numerous spots in the Afar Region where satellite trees or patches of *Prosopis* occur.

Moreover, the fractional cover map also helped identifying high-value areas that are at risk of invasion by *Prosopis*. For example, areas of nature reserves bordering *Prosopis* invaded rangeland should be monitored and young plants continuously removed. In regions where *Prosopis* occurs in the surroundings of protected areas and livestock is allowed to enter these areas, as it is currently the case for Awash National Park, measures may be needed to fence livestock away from *Prosopis* prior to entering the protected areas to prevent establishment of new satellite stands from defecated seeds which remain viable after gut passage^75^.

The rate of spread of *Prosopis* in the Afar Region and the strong indication that this species has not yet filled its environmental niche in the region call for consideration of a sustainable long-term management of this aggressive invader, also in remote areas. Experiences made with woody IAPS management suggest that a combination of biological control reducing reproductive output and physical control removing established trees is among the most promising and sustainable strategies, if not the only one, to reduce cover of dominant invasive tree species^22,72^. In Australia, the release of a biological control agent, the leaf-tying moth *Evippe* sp. (Lepidoptera: Gelechiidae), has resulted in very low growth and fertility rates of *Prosopis*^76^.

## Conclusions

Using *Prosopis* invasion in the Afar Region, Ethiopia, as a model system, our study suggests that the RF regression can help producing accurate fractional cover maps of IAPS that help explaining their current distribution and abundance as well as predicting future invasion patterns. The incorporation of environmental variables significantly improved the accuracy of the model and revealed that landscape structure variables, such as distance to rivers, road and villages, were more significant drivers of *Prosopis* distribution than climatic variables, suggesting that *Prosopis* is likely to continue spreading and increasing in abundance in the case study area if left uncontrolled. The knowledge gained does not only generate basic knowledge on IAPS invasion processes, it also provides support for the development and implementation of spatially explicit management strategies that may include different management interventions in different parts of the invaded range, and with different priorities. In particular, because of the high accuracy of our model, achieved partly through extensive sampling of field data, our approach allowed identification of numerous areas with low *Prosopis* cover, i.e. areas with early invasion stages that should be targeted for rapid response (removal of isolated patches at the invasion front) to prevent further spread of this IAPS in the case study region. We propose that the establishment and interpretation of fractional cover maps of IAPS should become a key component of IAPS management at the regional or national scale.

## Electronic supplementary material

supplementary or appendices
