# Supplementary material for: Modelling the current fractional cover of an invasive alien plant and drivers of its invasion in a dryland ecosystem
Source: Sci Rep. 2019 Feb 7;9:1576. doi: 10.1038/s41598-018-36587-7 (PMC6367408; doi:10.1038/s41598-018-36587-7)

**Modelling the current fractional cover of an invasive alien plant and drivers of its invasion in a dryland ecosystem**

Hailu Shiferaw^1,3*^, Urs Schaffner^2^, Woldeamlak Bewket^3^, Tena Alamirew^1^, Gete Zeleke^1^, Demel Teketay^4^, and Sandra Eckert^5^

^1^Water and Land Resource Centre, P.O. Box 3880, Addis Ababa, Ethiopia; and private P.O.Box 7985, Addis Ababa, Ethiopia.

^2^CABI, Rue des Grillons 1, CH-2800 Delémont, Switzerland.

^3^Department of Geography & Environmental Studies, Addis Ababa University: P.O.Box 1176, AAU, Ethiopia.

^4^Botswana University of Agriculture and Natural Resources (BUAN), Department of Crop Science and Production, Private Bag 0027, Gaborone, Botswana.

^5^Centre for Development and Environment, University of Bern, Hallerstrasse 10, CH-3012 Bern, Switzerland.

^*^corresponding author: Hailu Shiferaw; email: [Hailu2nd@gmail.com](mailto:Hailu2nd@gmail.com); [Hailushi31@yahoo.com](mailto:Hailushi31@yahoo.com)

**Appendices**

|  | | | | | | | | | | | | | | | | | | |
| --- | --- | --- | --- | --- | --- | --- | --- | --- | --- | --- | --- | --- | --- | --- | --- | --- | --- | --- |
| Evaluation parameter | Scenario 1 | | | Scenario 2 | | | Scenario 3 | | | | | | | | | | | |
|  | > 5 % input threshold:  Calibration vs validation | | | > 5 % input threshold: Calibration vs validation | | | > 5 % input threshold: calibration vs validation | | | > 20 % input threshold: calibration vs validation | | | > 40 % input threshold: calibration vs validation | | | > 60 % input threshold: calibration vs validation | | |
|  | 80/20 | 70/30 | 60/40 | 80/20 | 70/30 | 60/40 | 80/20 | 70/30 | 60/40 | 80/20 | 70/30 | 60/40 | 80/20 | 70/30 | 60/40 | 80/20 | 70/30 | 60/40 |
| Accuracy | **0.92** | 0.91 | 0.893 | 0.83 | 0.82 | 0.80 | 0.89 | 0.89 | 0.88 | 0.90 | 0.87 | 0.88 | 0.89 | 0.89 | 0.88 | 0.90 | 0.87 | 0.88 |
| Kappa-coefficient | **0.80** | 0.76 | 0.712 | 0.58 | 0.53 | 0.45 | 0.72 | 0.73 | 0.69 | 0.72 | 0.64 | 0.65 | 0.71 | 0.71 | 0.64 | 0.72 | 0.64 | 0.65 |
| AUC | **0.97** | 0.96 | 0.939 | 0.87 | 0.85 | 0.82 | 0.95 | 0.95 | 0.93 | 0.95 | 0.93 | 0.93 | 0.94 | 0.94 | 0.93 | 0.948 | 0.93 | 0.93 |
| Correlation | **0.81** | 0.80 | 0.762 | 0.64 | 0.59 | 0.53 | 0.78 | 0.77 | 0.76 | 0.75 | 0.71 | 0.72 | 0.76 | 0.77 | 0.73 | 0.752 | 0.70 | 0.71 |
| Threshold | **0.32** | 0.42 | 0.269 | 0.31 | 0.28 | 0.21 | 0.33 | 0.26 | 0. 26 | 0.23 | 0.20 | 0.33 | 0.32 | 0.38 | 0.26 | 0.233 | 0.20 | 0.33 |
| Variables removed | Red | NIR | SWIR | Red | Red | NIR | Relief | Rugged | Landform | Rugged | Relief | Rugged | Slope | Landform | Landform | Rugged | Rugged | Landform |
|  |  |  |  |  |  |  |  |  |  |  |  |  |  |  |  |  |  |  |

**Appendix 1**. Comparison of important variables for current distribution of *Prosopis* at different calibration and validation proportions for three scenarios: all 17 variables (scenario 1), only image reflectance variables (5 variables: scenario 2), and only environmental variables (12 variables: scenario 3). The variables removed from the final model from each scenario are also indicated. The first number is the proportion of samples used for model calibration (training data) while the second number is the proportion of samples used for validation (test data). Three different proportions (80/20, 70/30, and 60/40) were tested to assess how the different amounts of training and test data influence the performance and variance statistics of the models.

**Appendix 2**. Distribution and abundance of *Prosopis* invasion in the Afar Region by district.

| Zone | District name | District’s total | Invaded area | | Mean percent |
| --- | --- | --- | --- | --- | --- |
|  |  | area (ha) | _______________ | | fractional cover |
|  |  |  | ha | % | (range) |
| Zone 1 | Aysaita | 135,494 | 56,072 | 41 | 23 (0.39-88) |
|  | Afambo | 188,133 | 44,117 | 23 | 19 (0.39-66) |
|  | Dubti | 577,731 | 153,964 | 27 | 18 (0.39-99) |
|  | Mile | 476,566 | 108,396 | 22.5 | 17 (0.39-93) |
|  | Elidar | 1,309,923 | 117,408 | 10 | 11 (0.39-87) |
|  | Chifra | 329,172 | 16,132 | 4 | 6 (0.39-39) |
|  | Kori | 308,829 | 18,529 | 5 | 6 (0.39-20) |
| Zone 2 | Berahle | 715,492 | 2,848 | 0.4 | 1.9 (0.39-43) |
|  | Erebti | 245,713 | 2,093 | 1 | 1.3 (0.39-42) |
|  | Megale | 197,019 | 436 | 0.2 | 9 (0.39-37) |
|  | Afdera | 1,273,157 | 12,821 | 1 | 7 (0.39-40) |
|  | Dalul | 324,661 | 53 | 0.02 | 3 (0.39-10) |
|  | Ab Ala | 128,289 | 2 | 0.001 | 0.5 (0.39-8) |
| Zone 3 | Amibara | 391,701 | 123,120 | 31 | 32 (0.39-99) |
|  | Dulecha | 126,089 | 53,067 | 42 | 31 (0.39-96) |
|  | Argoba Special | 47,104 | 7 | 0.01 | 3 (1.8-55) |
|  | Awash Fentale | 104,215 | 39,730 | 38 | 31 (0.39-89) |
|  | Bure Mudaytu | 110,382 | 74,972 | 68 | 30 (0.39-89) |
|  | Gewane | 864,707 | 252,373 | 29 | 29 (0.39-99) |
|  | Koneba | 67,577 | - | - | - |
| Zone 4 | Teru | 365,860 | 86,792 | 24 | 23 (0.39-87) |
|  | Yalo | 182,064 | 15,369 | 8.44 | 18 (0.39-94) |
|  | Awra | 301,548 | 53,763 | 17.83 | 11 (0.39-83) |
|  | Gulina | 132,614 | 1,721 | 1.30 | 9 (0.39-66) |
|  | Ewa | 120,541 | 6,946 | 5.76 | 6 (0.39-31) |
| Zone 5 | Artuma | 37,424 | 2,707 | 7.23 | 24 (0.39-92) |
|  | Dewe | 106,005 | 14,243 | 13.44 | 17 (0.39-82) |
|  | Fursi | 128,390 | 26,593 | 20.71 | 12 (0.39-83) |
|  | Simurobi Gele'alo | 124,779 | 16,547 | 13.26 | 11 (0.39-53) |
|  | Telalak | 139,137 | 20,357 | 14.63 | 10 (0.39-77) |
| Area & fractional cover | | 9,510,974 | 1,172,918 |  | 12.33 % |

**Appendix 3**. Average weighted mean value (‘wtm’) of environmental correlates. Number above each plot indicates the mean of functional ranges of variables for *Prosopis* distribution as a driver variable. Y-axis for all plots are fitted values, and x-axis are different units depending on the datasets.


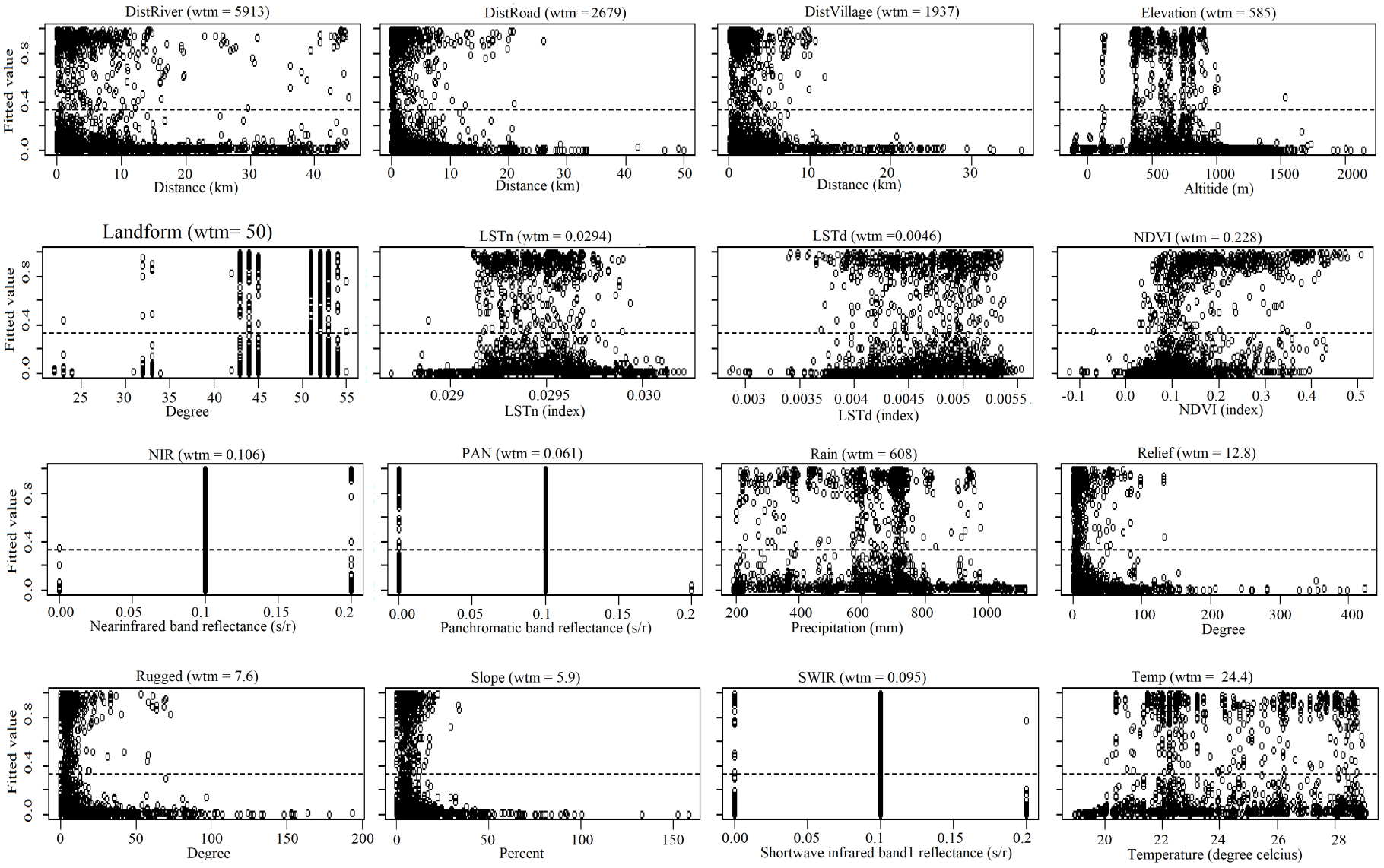

Supplement: Supplementary file 1 — supplementary or appendices [file 41598_2018_36587_MOESM1_ESM.docx]
